# Applying targeted gene hybridization capture to viruses with a focus to SARS-CoV-2

**DOI:** 10.1016/j.virusres.2023.199293

**Published:** 2023-12-16

**Authors:** Andres Ceballos-Garzon, Sophie Comtet-Marre, Pierre Peyret

**Affiliations:** Université Clermont Auvergne, INRAE, MEDiS, 63000, Clermont-Ferrand, France

**Keywords:** Hybridization capture, Sequence enrichment, Probes, Next-generation sequencing, SARS-CoV-2, COVID-19

## Abstract

Although next-generation sequencing technologies are advancing rapidly, many research topics often require selective sequencing of genomic regions of interest. In addition, sequencing low-titre viruses is challenging, especially for coronaviruses, which are the largest RNA viruses. Prior to sequencing, enrichment of viral particles can help to significantly increase target sequence information as well as avoid large sequencing efforts and, consequently, can increase sensitivity and reduce sequencing costs. Targeting nucleic acids using capture by hybridization is another efficient method that can be performed by applying complementary probes (DNA or RNA baits) to directly enrich genetic information of interest while removing background non-target material. In studies where sequence capture by hybridization has been applied to the severe acute respiratory syndrome coronavirus 2 (SARS-CoV-2) virus, most authors agree that this technique is useful to easily access sequence targets in complex samples. Furthermore, this approach allows for complete or near-complete sequencing of the viral genome, even in samples with low viral load or poor nucleic acid integrity. In addition, this strategy is highly efficient at discovering new variants by facilitating downstream investigations, such as phylogenetics, epidemiology, and evolution. Commercial kits, as well as *in-house* protocols, have been developed for enrichment of viral sequences. However, these kits have multiple variations in procedure, with differences in performance. This review compiles and describes studies in which hybridization capture has been applied to SARS-CoV-2 variant genomes.

## Highlights

• We describe gene capture by hybridization approach coupled to high throughput sequencing targeting viruses as a strategy to reveal their genetic diversity in various biological samples.
• We compared viral genomes enrichment efficiency results through various probe sets description.
• We compiled studies results applied in SARS-CoV2 genomes enrichment.
• We indicate limits and advantages of sequence capture by hybridization compared to current classical molecular methods.

## 1 Introduction

Over the past 20 years, three coronaviruses that originally came from animals, namely, the severe acute respiratory syndrome coronavirus (SARS-CoV), the Middle East respiratory syndrome coronavirus (MERS-CoV), and the severe acute respiratory syndrome coronavirus 2 (SARS-CoV-2) have crossed over to infect humans. This has led to significant public health problems and global emergencies (Zhou et al., 2020). CoVs are a highly diverse family of enveloped positive-sense single-stranded RNA viruses, and their 5′-capped RNA molecules range from 26 to 32 kb and contain at least 6 open reading frames (ORFs) (Perlman and Netland, 2009). The SARS-CoV-2 genome consists of approximately 29,800 nucleotides arranged into ∼12 ORFs. The majority of the genome comprises ORF1a and ORF1b, which encode 16 different non-structural proteins (nsp1-nsp16). The remaining ORFs encode structural proteins (i.e., spike, S; envelope, E; membrane, M; nucleocapsid, N) and accessory proteins (i.e., ORF3a,b,c,d; ORF6; ORF7a,b; ORF8a; ORF9b,c; ORF10), which play important roles in pathogenesis and are useful for diagnosis and treatment strategies (Ellis et al., 2021; Malik et al., 2021).

Since SARS-CoV-2, the causative agent of coronavirus disease 2019 (COVID-19), was declared a global pandemic, approximately 670 million people have tested positive worldwide, with more than 6.9 million deaths (https://www.worldometers.info/coronavirus/coronavirus-death-toll/, October 2023). As accurate viral detection is a starting point to contain and follow the COVID-19 pandemic, the response of the diagnostics industry was overwhelming. Today, there are more than 1000 brands of SARS-CoV-2 diagnostic tests available on the market, with more in the pipeline (Peeling et al., 2022). Diagnosis of COVID-19 is mainly based on evaluation of signs and symptoms, with confirmation by reverse transcription-quantitative polymerase chain reaction (RT‒qPCR) for qualitative and quantitative detection of viral nucleic acids from different samples, with nasopharyngeal swabs being the most commonly used sample (Maia et al., 2022). Other nucleic acid-based assays include loop-mediated isothermal amplification (LAMP), real-time LAMP (RT-LAMP), multiple cross displacement amplification (MCDA), recombinase-aided amplification (RPA) and clustered regularly interspaced short palindromic repeats (CRISPR)-specific high-sensitivity enzymatic reporter unlocking (SHERLOCK) (Ebrahimi et al., 2022; Ghosh et al., 2022; Maia et al., 2022). Furthermore, serological methods such as enzyme-linked immunoassays for viral antibody and antigen detection, imaging tests (e.g., computed tomography and positron-emission tomography), and nanoparticle-based detection have been applied for COVID-19 diagnosis (Gupta et al., 2020; Maia et al., 2022).

RT-qPCR is the reference method for diagnosis, is widespread and commonly used, provides rapid positive/negative results, and has been useful for the detection and tracking of worrying variants. However, it does not provide information on the genetic sequence of the virus, possible infection by two variants or any information on virulence. In general, a sequencing system to support PCR detection is beneficial to ensure that the test remains sensitive and to be able to adapt the method to new variants (Illumina, 2022). Sequencing can provide unquestionable evidence for detection and evolution of SARS-CoV-2; for instance, it has been used to identify new emerging strains such as alpha (B.1.1.7), beta (B.1.351), gamma (P.1), delta (B.1.617.2) and omicron (B.1.1.529) (Manjur et al., 2022). In addition, genomic surveillance is a useful tool for identifying the dynamics of viral spread, monitoring viral changes and adaptation, ensuring accurate diagnosis, guiding research on therapeutic targets and refining public health strategies (Gire et al., 2014).

Next-generation sequencing (NGS) has been applied to various fields in virology, e.g., genome characterization and evolution, virome diversity exploration, target identification for the development of therapeutics, and diagnosis and identification of new viruses (Radford et al., 2012). Nevertheless, the low pathogen-to-nucleic acid ratio in the host is often an obstacle to detecting/reconstructing virus genomes. To overcome these limitations, enrichment approaches such as amplicon sequencing and gene capture by hybridization followed by NGS sequencing have been developed. These methods have led to an increase in thousands of folds of sequences of interest compared to the genomic background, ensuring that they are predominantly represented in the sequenced DNA/RNA while avoiding large sequencing efforts and fastidious bioinformatics treatments (Wylezich et al., 2021).

Amplicon sequencing utilizes PCR to amplify genomic targets before sequencing. It is relatively simple to implement and sensitive; however, the nature of PCR limits the length of the amplified genomic regions to a few kb in the best scenario and requires the presence of specific and conserved priming sites in the sequences targeted (Kuchinski et al., 2022). In contrast, the hybridization capture method avoids PCR biases while allowing for efficient recovery of targets. In addition, large genomic fragments can be recovered to assemble complete genomes with high sequencing coverage, facilitating downstream bioinformatics analyses such as phylogenetics, evolution, epidemiology and drug resistance surveys (Gasc and Peyret, 2018, 2017; No et al., 2019; Quick et al., 2017). Moreover, the high sensitivity of hybridization enables detection of viral genomes in samples negative by RT‒qPCR (Pipoli da Fonseca et al., 2022).

**Fig. 1:**
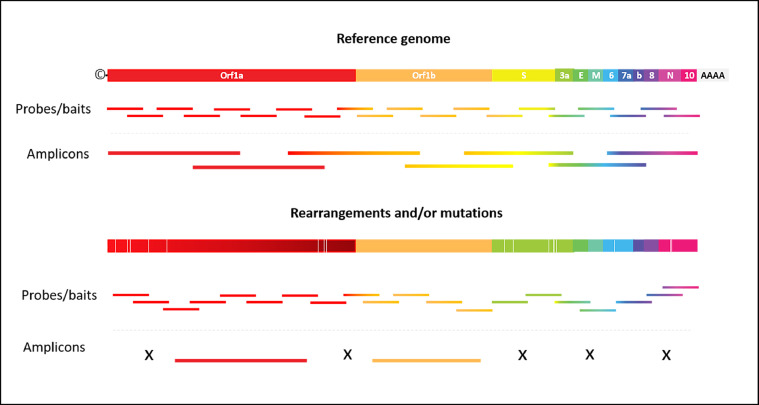
Nucleic acid enrichment by hybridization capture vs. amplicon approach. X represents a missed genomic region.

Both hybridization capture and amplicon sequencing approaches can effectively enrich regions of known sequences. However, only hybridization capture can recover target sequences that have significant rearrangements and/or mutations from the reference sequence used for probe design (Fig. 1). Bearing in mind the above, this review outlines the core concept of gene capture through hybridization combined with sequencing. Additionally, we compile existing commercial solutions and in-house developed methods for SARS-CoV-2 surveys and their use in the context of the COVID-19 pandemic.

## 2 Hybridization capture, principle and applications

**Fig. 2:**
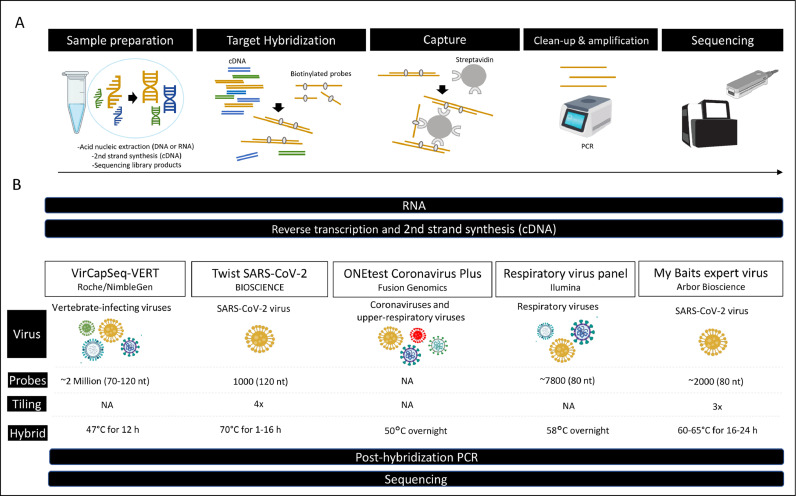
Workflow for SARS-CoV-2 capture hybridization. A) Schematic representation of hybridization capture for virus detection. B) Overview of five commercial kits for SARS-CoV-2 capture. NA, no information available. Hybrid, temperature, and time of hybridization.

Basically, hybridization capture is based on the use of probes that are complementary to the target sequence, acting like a hook to enable specific enrichment of genes or genomes of interest (Ribière et al., 2016). Hybridization capture-based target enrichment can be performed in solution or on a solid support (also called solid phase) using immobilized probes. However, solution capture is the most commonly used (Gaudin and Desnues, 2018). In the latter approach, free DNA or RNA probes are biotinylated to enable selection of targeted fragment-probe heteroduplexes using streptavidin-coupled magnetic beads. In general, sequencing libraries derived from metagenomic DNA or RNA are used as a sample, but nuclear extracts can also be used. Non-targeted fragments are removed from the liquid phase by repeated washes, and the targeted fragments are eluted from the beads. Once eluted, these fragments can be amplified for subsequent sequencing or sequenced directly (Gasc et al., 2016; Wylezich et al., 2021). Bioinformatic analyses enable reconstruction of the full genome and identification of mutations (Fig. 2A).

Many parameters such as probe length, melting temperature (Tm), %GC, complexity or still potential cross-hybridizations evaluation (percent similarity and identity stretch between probe and non-targeted sequences) should be taken into account to ensure the selection of the probes offering the best compromise between specificity and sensitivity. Long probes (>50 mers) show high sensitivity (efficient target recovery in complex samples), also allowing mismatches to target close sequence variants but a lower specificity also enrich off-targets. As DNA:DNA hybrids are less stable and have a weaker hybridization efficiency than RNA:DNA and RNA:RNA complexes, RNA baits might be preferable (Chein and Davidson, 1978). However, care must be taken when working with RNA baits, which are not as stable as DNA probes (Wylezich et al., 2021). Probes are designed across a target region to achieve the desired tiling density, which refers to the extent of coverage of the target region by probes. For example, 1× tiling density means that the probes cover the region of interest one time. In contrast, 2× tiling density means that the region of interest is covered twice using a series of overlapping probes (Munyuza et al., 2022). Unlike PCR primers (18–20 nt), the probes used for capture hybridization are typically 80–120 nucleotides in length; thus, sequence divergence and nucleotide mismatches are more tolerated (Gasc and Peyret, 2018, 2017; Kuchinski et al., 2022). In studies that focus on highly diversified viruses, probe design must be carefully considered in an attempt to include all subtypes and groups. To design probes for variable sequences such as those present in different subtypes, one strategy is to first design probes based on a consensus sequence and then design probes that cover the variable regions for each subtype to be covered. Alternatively, probes can be designed to be subtype-specific rather than including all subtypes (Miyazato et al., 2016; Munyuza et al., 2022). Furthermore, design of exploratory probes may be considered to anticipate genetic variations using degenerate consensus probes that combine known genetic diversity. These probes would hybridize with described as well as undescribed but potentially existing sequences because they maintain the structure of the gene, as previously described by our group (Gasc and Peyret, 2018, 2017). In addition, a virtually unlimited number of probes can be employed for hybridization, allowing simultaneous capture of thousands to millions of target sequences. Consequently, such probes/baits are suitable and useful for applications in which diverse and/or hypervariable viruses such as SARS-CoV-2 are targeted (Wylezich et al., 2021).

Target isolation by hybridization and subsequent enrichment has proven to be effective in genome and exome resequencing studies, allowing for selection of deep sequence-specific regions within the human genome (Hodges et al., 2007). This method has been employed to enrich for herpesvirus genomes from a range of clinical samples, including saliva, blood, viral vesicles, cerebrospinal fluid and tumor cell lines. In their study, the authors demonstrated the efficacy of the method by sequencing 13 highly cell-associated human herpesviruses, in which full-length genomes were reconstructed at high coverage depth (Depledge et al., 2011). Subsequently, the technique has been widely used to study a wide range of viruses, including those of clinical importance, such as hepatitis C virus (HCV), varicella zoster virus, and human immunodeficiency virus (HIV), among others (Depledge et al., 2011; Metsky et al., 2017; Munyuza et al., 2022; Thomson et al., 2016), and more recently, SARS-CoV-2 and monkeypox viruses (Müller et al., 2021; Rehn et al., 2021).

## 3 In house and commercial kits for SARS-CoV-2 capture by hybridization

Several turnkey solutions have been marketed, including sequencing library preparation, hybridization capture kits and probe panels, some of which are dedicated only to SARS-CoV-2 genome enrichment (MyBaits SARS-CoV-2 panel, KAPA HyperCap SARS-CoV-2 and Twist Bioscience SARS-CoV-2 Research panel), whereas others enable enrichment of several respiratory virus genomes (Illumina respiratory panel v1 and v2, Illumina Viral Surveillance panel, Respiratory Pathogen ID/AMR Enrichment Panel Kit, VirCapSeq-VERT Capture Panel, Twist Respiratory Virus Research Panel, ONETest™ Coronaviruses Plus). Examples of studies in which these kits and non-commercial probes have been used are described in Table 1. In addition, a list of viruses targeted by each kit is provided in Table S1. It is important to emphasize that all these kits are exclusively designed for research purposes. Notably, there is no established standard protocol for the target enrichment steps, as each technology employs its proprietary workflow, often influenced by factors such as the composition of the hybridization buffer and the characteristics of the probes (Fig. 2B). Detailed protocols with specificity, sensitivity and reproducibility values for some procedures tested are detailed in Rehn et al. (2021).

**Table 1:** Examples of studies using target enrichment by hybridization capture on SARS-CoV-2.

Approaches employed in the article	Hybrid capture-based kit/protocol	Sample	Reads	% mapped reads SARS-CoV-2	Coverage SARS-CoV-2	Sequencing device (captured library PCR amplification)	Reference
Capture Amplicon	1) Respiratory virus panel Illumina 2) Twist Bioscience SARS-Cov-2 panel	NPS and BAL	1) 0.4–4.7 M 2) 0.2–15 M	0–95 % (mean 74 %) 0–99 % (mean 51 %)	NA	MiSeq	(Klempt et al., 2020)
Capture Amplicon	2019-nCoVirus DNA/RNA Capture Panel (BOKE, Beijing, China). 506 probes (ssDNA 120 nt) tiling 2×	NPS, virus from cell culture, sputum, and anal swabs	1.5–1.7 M	0–5 %	Near-complete sequencing coverage >95 %	MGISEQ-2000	(Xiao et al., 2020)
Capture	Development of 502 single-stranded biotin-labelled DNA probes (120 nt).	Virus from cell culture and NPS	13–97 M	NA	Near-complete sequencing coverage >90 %	MGISEQ-2000 (13–17 cycles)	(Wen et al., 2020)
Capture Amplicon	myBaits Expert Virus SARS-CoV-2 panel	NPS	0.2–1 M	1–89 % (mean 59 %)	NA	MiSeq	(Nasir et al., 2020)
Capture Amplicon	Respiratory virus panel Illumina	NPS	0.1–40 M	NA	Near-complete sequencing coverage >90 % in 91 % of specimens	NextSeq 550 (25 cycles)	(Charre et al., 2020)
Capture	Twist Bioscience SARS-Cov-2 panel	NPS	0.3–45 M	(mean 50 %)	Near-complete sequencing coverage >95 % in specimens with Ct < 30	NextSeq 550	(Nagy-Szakal et al., 2021)
Capture	Twist Bioscience SARS-Cov-2 panel	NPS	1–805 M	0–80 % (mean 64 %)	Full-length genomes were obtained from 17 (38 %) of the 45 capture-enriched samples (Ct 17–39)	NovaSeq 6000 (12–20 cycles)	(Doddapaneni et al., 2021)
Capture	VirBaits (177,471 RNA baits) targeting 35 epizootic and zoonotic viruses.	NPS	0.04–09 M	3–100 % (mean 46 %)	Near-complete sequencing coverage >88 % in 42 % of specimens	Ion Torrent S5XL	(Wylezich et al., 2021)
Capture Amplicon	ONEtest Fusion Genomics	NPS and endotracheal aspirates	0.4–6 M	0–100 % (mean 23 %)	Near-complete sequencing coverage >90 % in 64 % of specimens	NextSeq 500 (20 cycles)	(Zhan et al., 2021)
Capture	1) VirCapSeq-VERT 2) Twist Respiratory Virus Research Panel	NPS	1) 982 M 2) 747 M	(mean 0.3 %) (mean 2.4 %)	Near-complete sequencing coverage >90 % in 95 % of specimens with Ct < 30 (Twist). VirCapSeq did not achieve > 90 % coverage even at 1× depth for all samples, except two with Ct < 20	NovaSeq 6000 (20 cycles)	(Kim et al., 2021)
Capture	2019-nCoVirus DNA/RNA Capture Panel (BOKE, Beijing, China.	NSP, throat swabs, sputum, gastric mucosa, urine, plasma, anal swabs, and feces	1000 M	(mean 0.4 %)	Complete genomes from the clinical samples with at least 60-fold sequence coverage	MGISEQ-2000	(Wang et al., 2021)
Capture	1) Respiratory Panels v1 and v2 Illumina 2) myBaits SARS-CoV-2 panel 3) Twist Bioscience SARS-CoV-2 panel 4) Twist respiratory panel	virus from cell culture	1) 0.3–0.4 M 2) 1 M 3) 0.5 M 4) 0.5 M	(mean 29 %) (mean 5 %) (mean 67 %) (mean 49 %)	Complete sequencing coverage (Study comparing capture enrichment panels)	MiSeq (12–16 cycles)	(Rehn et al., 2021)
Capture	Respiratory virus panel Illumina V2	NPS and saliva	NA	NA	Near-complete sequencing coverage >90 % in 60 % of specimens	NextSeq 550	(Sahajpal et al., 2021)
Capture	myBaits Virus SARS-CoV-2 panel	NPS	0.04–8 M	NA	Near-complete sequencing coverage >90 % in 86 % of specimens	NextSeq 500 (8–16 cycles)	(Hartley et al., 2021)
Capture	VirCapSeq-VERT	NPS, urine, BAL and blood	Illumina 63 M MinION 1.2 M	1.2 % 0.2 %	Illumina 96 % MinION 74 %	NextSeq500 & MinION	(Pogka et al., 2022)
Capture	Custom probe panel (20,000 hybridization probes)	Rectal and oral swabs (bats)	0.01–7 M	(mean 14 %)	Near-complete sequencing coverage >94 % in 90 % of specimens	MiSeq (25 cycles)	(Kuchinski et al., 2022)
Capture Amplicon	SureSelect CD Pan Human Coronavirus Panel (Agilent)	NPS	NA	NA	99 % (Ct < 20)	NovaSeq 6000	(Gerber et al., 2022)
Capture	1) Illumina V2 2) Twist Bioscience (Pan viral & SARSCoV2) 3) myBaits Virus SARS-CoV-2 panel	NPS	1) 0.7–31 M 2) 1.5–26 M/0.5–5.5 M 3) 0.1–7.7 M	1) 0–75 % 2) 0–1.8 %/0–95 % 3) 0–94 %	1) mean 89 % 2) mean 25 %/90 % 3) mean 90 % (Ct 26–39)	NextSeq 500	(Pipoli da Fonseca et al., 2022)

NPS: nasopharyngeal swabs; BAL: bronchoalveolar lavage fluid; M: millions; NA: no data.

The VirCapSeq-VERT Capture Panel (Roche, Pleasanton, CA, USA) enables detection of viral sequences in complex sample backgrounds (e.g., serum, blood, and tissue). The multiplexed nature of the system allows for both simultaneous identification and genetic characterization of a comprehensive set of known vertebrate viruses (207 viral taxa), with strong emphasis on retroviruses, their genetic variants, and novel related viruses. The panel was constructed using a database of 342,438 representative sequences covering all virus sequence records, excluding bacteriophages. VirCapSeq-VERT enables background human DNA reduction and 100–10,000-fold enrichment of viral reads compared to other enrichment procedures, such as nuclease treatment or RiboZero rRNA depletion (Briese et al., 2015). However, this kit has been discontinued. Roche has proposed KAPA HyperCap SARS-CoV-2 Panel, which targets 99.7 % of 183 publicly available SARS-CoV-2 genomes and 100 % of the reference genome. The kit achieves 1× coverage of ∼97 % of the SARS-CoV-2 genome down to 1000 viral copies and captures genomic sequences from as few as 10 viral copies with a hybridization time as short as one hour (https://sequencing.roche.com).

On the other hand, Respiratory Virus panel V2 (RVOP v2, cat number 20044311, Illumina) targets ∼40 common respiratory viruses, including SARS-CoV-2, through ∼7800 probes. In addition, Illumina commercialized the Viral Surveillance panel, which enriches the viral genomes of 66 viruses considered to be of high public health risk, such as SARS-CoV-2, influenza, monkeypox virus and poliovirus, from a range of host and environmental samples, including wastewater. Interestingly, Viral Surveillance Panel follows the same library preparation protocol as Illumina Respiratory Virus Oligo Panel (Illumina, n.d.).

The Twist SARS-CoV-2 Research panel (Twist Bioscience, San Francisco, CA, USA) targets approximately 30 kb of the genome of SARS-CoV-2 using approximately 1000 probes. In a protocol of 16-h hybridization in a 2-day target enrichment workflow, it generates enriched DNA libraries for sequencing using Illumina NGS systems (“Twist SARS-CoV-2 Research Panel | Twist Bioscience,” n.d.). However, using Twist's fast hybridization and multiplexed capture workflow, libraries ready for high-throughput sequencing can be constructed from clinical specimen extracts in less than 6 h. The same manufacturer (Twist Bioscience) offers Twist Respiratory Virus Research Panel, designed to capture 29 common human respiratory viruses, including CoVs, influenza virus, adenoviruses, bocavirus (hBoV), enterovirus, human rhinoviruses (HRV), metapneumovirus, measles morbillivirus (MeV), mumps virus (MuV), parainfluenza viruses (hPIV), rubella virus, and respiratory syncytial virus (RSV). The 41,047 probes in this panel allow for separating SARS-CoV-2 from other respiratory viruses, both influenza- and non-influenza-related (Briese et al., 2015); simultaneous identification of viral co-infections, as recently described in an Australian study, can also be achieved (Kim et al., 2021).

ONETest™ Coronaviruses Plus is based on a proprietary target capture NGS platform developed by Fusion Genomics Corp. (Burnaby, BC, Canada). This assay captures human and animal CoVs as well as known upper respiratory viruses (Fusion Genomics, 2021; Zhan et al., 2021). ONETest Coronaviruses Plus allows for capture of the entire SARS-CoV-2 genome from total nucleic acid extracts, enabling an ∼10,000-fold increase in viral reads**.** In a proof-of-concept study comparing ONETest with the amplicon-based protocol from ARTIC Network (https://artic.network/), ONETest yielded a complete or near-complete SARS-CoV-2 genome more often than the ARTIC approach, allowing for SARS-CoV-2 variants to be tracked and monitored (Briese et al., 2015). The company is also developing an ultrafast approach enabling sample-to-sequencing in two hours (ONETest RAPID assay).

Finally, the MyBaits Expert Virus—SARS-CoV-2 panel (Arbor Biosciences, Ann Arbor, MI, USA) provides a targeted solution to enrich for only the SARS-CoV-2 genome; the supplier also offers customized probes. Unlike the above-mentioned kits (DNA probes), this kit uses biotinylated ssRNA baits. These baits are 80 nucleotides long and designed using all complete and partial sequences of the SARS-CoV-2 genomes available at NCBI as of 31 January 2020. The myBaits panel covers the entire viral genome, showing >99.9 % coverage in samples with ∼10^6^ viral genome copies (Nasir et al., 2020; “Targeted sequencing of sars-cov-2: swift rna library kit and arbor biosciences mybaits expert virus panel”, 2020). The kit does not include a library preparation step.

Interestingly, custom probe panels dedicated to specific targets can be used with these kits and with Agilent SureSelect capture platforms and Roche NimbleGen SeqCap EZ platforms (Shih et al., 2018; Sulonen et al., 2011). Most of them offer a custom probe design service. For instance, HUBDesign software was validated for virus probe design by generating probe sets targeting the breadth of CoV diversity. The authors captured SARS-CoV-2 (400 probes) in a human RNA background, demonstrating significant enrichment (97-fold). The probes were synthesized through the Ann Arbor Biosciences myBaits custom DNA-Seq program (Dickson et al., 2021).

## 4 Target enrichment performance

Although all the kits described above aim to enrich for the SARS-CoV-2 virus, they have multiple variations in their procedures and probe panels (pan-viral or SARS-CoV2 specific) and thus lead to differences in performance on various samples. For instance, Rehn et al. described for the first time a combined approach of droplet digital PCR (ddPCR) and NGS to evaluate five commercially available sequence capture bait panels targeting SARS-CoV-2 (i.e., Illumina respiratory panels v1 and v2, MyBaits SARS-CoV-2 panel, Twist Bioscience SARS-CoV-2 panel and respiratory panel) to determine the most sensitive and most efficient one on virus cell culture samples. Their results showed that all tested bait panels were able to bind SARS-CoV-2, though they displayed differences in sensitivity and enrichment capacity. To evaluate the sensitivity and capture efficiency of the five different bait panels, primers targeting ORF1a were used to quantify the presence of SARS-CoV-2-specific library fragments; human ubiquitin C (UBC) was used to detect non-targeted human DNA. The two Illumina panels showed the highest ORF1a/UBC ratio, followed by the Twist Bioscience SARS-CoV-2 panel, the Twist Bioscience respiratory panel, and the myBaits SARS-CoV-2 panel. Furthermore, to analyze the minimum number of reads needed to retrieve a full-length SARS-CoV-2 genome with a coverage of at least 20-fold, the authors found that use of the SARS-CoV-2 panel from Twist Bioscience resulted in the lowest number of reads needed to recover a full-length SARS-CoV-2 genome (from nearly 6000 to approximately 5,000,000 reads depending in C_T_ values of the input pools being respectively 19.3 and 33.4). Overall, the SARS-CoV-2 panel of Twist Bioscience was the most sensitive and most efficient capture panel, followed by the respiratory panel v2 from Illumina, its progenitor respiratory panel v1, and the MyBaits SARS-CoV-2 panel (Rehn et al., 2021). More recently, Pipoli da Fonseca et al. also tested the efficacy of five commercial probe panels from three manufacturers, two from Illumina (Respiratory Virus Oligo Panel, ssDNA, version v1 & v2), two from Twist Bioscience (Pan-Viral Panel and SARS-CoV-2 Panel, dsDNA), and one from Arbor Bioscience (SARS-CoV-2 Mybaits Panel, ssRNA), for detection of the SARS-CoV-2 genome from nasopharyngeal swabs with low viral abundance. Two of the five panels contained only probes specific to the SARS-CoV-2 genome (SARS-CoV-2 Panel, Twist & My baits, Arbor), whereas the other three were pan-virus (Respiratory Virus Oligo Panel, Illumina & Pan viral panel, Twist). The results obtained by the pan-viral panels in terms of capturing SARS-CoV-2 showed that the Illumina respiratory virus panel appears to be the best multi-viral panel for capturing the entire viral genome from patient samples. However, the results suggested that both panels are able to capture high-abundance targets but fail to capture lower-abundance targets, with a high percentage of off-target reads from host DNA. Regarding SARS-CoV-2-only panels, the authors concluded that both were very effective. The Arbor panel showed the highest percentage of on-target reads. Its effectiveness was especially evident for samples with lower viral loads (Ct > 28). However, the authors noted that this panel requires a double-capture protocol. Interestingly, when a single-capture protocol was used for both panels, the Twist panel showed a higher percentage of on-target reads than the Arbor panel. On the other hand, when a metagenomics analysis of pre-captured samples (∼21 M reads for each sample) was performed, no reads matched the SARS-CoV-2 genome, suggesting that either the samples did not contain any SARS-CoV-2 sequences or that the sequencing depth was insufficient (mapped reads: 0.01–5 M) to detect low-abundance SARS-CoV-2 in the samples (Pipoli da Fonseca et al., 2022). Hence, the important role of hybridization capture, mainly in samples with high Ct values, becomes evident.

Another published study comparing capture methods from nasopharyngeal swabs of patients diagnosed with COVID-19 evaluated the performance of three commercial preparation kits using both amplicon and hybridization capture (Twist Bioscience) approaches. According to their results, the Ct value was the major predictor of sample outcome in regard to achieving 20× coverage, regardless of the type of workflow. In samples with Ct values ranging from 11 to 22, the negative correlation of median coverage did not show such a significant trend. Enrichment-based approaches required a higher number of reads (min. 109,624 PE reads) to reach the optimal coverage limit than the amplicon approach (75,684 PE reads). However, the evenness of coverage was better in both capture-based protocols than in amplicon-based protocols. Overall, the authors reported better performance (greater uniformity in coverage and lower false-positive rates) with capture enrichment compared to amplicon enrichment (Klempt et al., 2020).

Kim et al. assessed respiratory viral co-infections among SARS-CoV-2-positive patients in Australia through respiratory virome characterization using pan-viral hybrid capture (VirCapSeq) and Twist Respiratory Virus Research Panel. In total, 8 % of patients were coinfected with rhinovirus or influenza virus. Twist capture demonstrated high breadth and depth of coverage, achieving near-complete reconstruction (>90 % coverage, >tenfold depth) of the SARS-CoV-2 genome in 95 % of specimens with Ct < 30, sufficient for downstream analysis of single-nucleotide variants, indels and structural variations. VirCapSeq did not achieve >90 % coverage at 1× depth for all samples, except for two, with Ct < 20 (Kim et al., 2021).

Three additional studies used capture probe sets for enrichment by hybridization of the SARS-CoV-2 genome from 3 (Wen et al., 2020), 8 (Xiao et al., 2020), and 45 (Doddapaneni et al., 2021) patient samples. Wen *et al*. designed a set of 502 (120 nt) biotin-labelled single-stranded DNA enrichment probes (2× tiling); use of capture allowed them to obtain a nearly complete SARS CoV-2 genome and to identify variants in specimens with low concentrations of viral particles (Ct > 32) (Wen et al., 2020). On the other hand, Xiao et al. applied multiplex PCR amplicon-based (amplicon) and hybrid capture-based (capture) sequencing (BOKE bioscience, capture panel), as well as ultra-high-throughput metatranscriptomic sequencing, to retrieve complete genomes and inter-individual and intra-individual variations in SARS-CoV-2 from serial dilutions of a cultured isolate and eight clinical samples covering a range of sample types and viral loads. They found that both amplicon and capture methods effectively enriched the SARS-CoV-2 content from clinical samples but that the enrichment efficiency of amplicon outperformed that of capture in more difficult samples. Moreover, capture by hybridization was not as accurate as metatranscriptomics and amplicon approaches in identifying between-sample variations (inter-individual and intra-individual). However, the amplicon method was not as accurate as the other two in investigating within-sample variations, suggesting that amplicon sequencing is not suitable for studying virus‒host interactions and viral transmission, which heavily rely on intra-host dynamics (Xiao et al., 2020). The BOKE capture panel contains 506 biotin-modified 120 nt ssDNA probes with a 2× tiling design (https://bokebio.com/rna-virus-captures-solutions/).

Regarding the Doddapaneni et al. study, the authors demonstrated use of capture enrichment (Twist Bioscience panel) to obtain full-length genome and viral transcriptional sub-regions generated during viral replication from patient samples without the need for a culture stage, allowing measuring transcription (enriching for genomic and transcriptional RNA). In samples with higher viral loads (Ct < 30), complete genomes were generated. Furthermore, bioinformatics analysis revealed regions of differential transcriptional activity among samples. Mixed allelic frequencies along the 20 kb ORF1ab gene in one sample suggested the presence of a defective viral RNA species subpopulation maintained in a mixture with functional RNA in one sample (Doddapaneni et al., 2021).

Zhan et al. introduced a target capture next-generation sequencing methodology, ONETest Coronaviruses Plus, to sequence the SARS-CoV-2 genome. They expanded the ONETest probe set (QuantumProbesTM; http://www.fusiongenomics.com/onetestplatform/), which originally targeted non-SARS-CoV-2 respiratory pathogens. The authors applied ONETest Coronaviruses Plus for 70 respiratory samples in which SARS-CoV-2 had been detected by a RT-PCR assay. For 48/70 samples, they also applied the ARTIC protocol. Of the 70 ONETest libraries, 45 (64 %) had a near-complete sequence (>29,000 bases and >90 % covered by >9 reads). For the 48 ARTIC amplicon libraries, 25 (52 %) had a nearly complete sequence. In samples in which both ONETest and ARTIC produced nearly complete sequences, the assigned lineages were identical. The authors concluded that as a target capture method, ONETest is less prone to loss of sequence coverage than amplicon methods and can therefore more frequently provide complete genomic information for tracking and monitoring SARS-CoV-2 variants (Zhan et al., 2021).

Gerber et al. compared the performance of three target enrichment methods, two multiplex amplification methods (Illumina COVIDSeq test and CleanPlex, Paragon genomics) and one hybridization capture method (Agilent SureSelect), using nasopharyngeal swabs from infected individuals. The authors described that each method has some advantages, such as high mapping rate (CleanPlex and COVIDSeq) or absence of systematic variant calling error (SureSelect) as well as limitations such as sub-optimal uniformity of coverage (CleanPlex), high cost (SureSelect, 127.57 USD/library not including RNA extraction) and supply shortages (COVIDSeq). Nevertheless, all three methods yielded an excellent breadth of coverage, greater than 99 % for all samples (Ct < 20). SureSelect alone provided coverage in the first and last few dozen nucleotides of the viral genome, which are not included in the primer design of either amplicon-based method (Gerber et al., 2022). SureSelect CD Pan Human Coronavirus Panel (ref 5191-6838, an Agilent customer probe design not validated by Agilent) covers all positions of the SARS-CoV-2 genome as well as of all other human coronaviruses.

Other gene capture by hybridization approaches have been used for enrichment of the SARS-CoV-2 viral genome. For instance, the generalized version of the RNA-mediated oligonucleotide Annealing Selection and Ligation with next-generation DNA sequencing (RASL-seq) assay, called “capture RASL-seq” (cRASL-seq), has been used for enrichment of SARS-CoV-2. cRASL-seq was highly sensitive (down to ∼1–100 PFU/mL). Importantly, cRASL-seq analysis of nasopharyngeal swab specimens from COVID-19 patients does not involve nucleic acid purification or reverse transcription, steps that introduce supply bottlenecks into standard assay workflows. Additionally, the protocol enables fast direct genotyping of SARS-CoV-2 polymorphisms across the entire genome, which can be used for enhanced characterization of transmission chains at the population scale and detection of viral clades with higher or lower virulence. cRASL-seq has limitations. Unlike unbiased metagenomic sequencing, target detection by cRASL-seq requires *a priori* selection of target organisms and knowledge of significant genomic variants (Credle et al., 2021).

Efforts to contain the spread of SARS-CoV-2 have stimulated the need for reliable, timely and cost-effective diagnostic methods that can be applied to a large number of individuals. Bokelmann et al. developed a point-of-care test using hybridization capture named Cap-iLAMP (capture and improved loop-mediated isothermal amplification), combining hybridization capture-based RNA extraction of gargle lavage samples with an improved colorimetric RT-LAMP assay and smartphone-based color scoring. In contrast to RT‒qPCR approaches, no complicated equipment, such as qPCR machines, is needed. Cap-iLAMP enables detection of SARS-CoV-2-positive samples in less than one hour. Although this is a merely diagnostic approach that targets only specific genes (Orf1a, N) across the SARS-CoV-2 genome, it demonstrates potential use of capture by hybridization in other settings (Bokelmann et al., 2021).

Overall, the kits that have been most widely used are as follows: Respiratory virus panel Illumina, Twist Bioscience SARS-CoV-2 panel and myBaits Expert Virus SARS-CoV-2 panel. All of them allow for efficient SARS-CoV-2 genome enrichment, facilitating complete or near-complete reconstruction of the viral genome, even in samples with low viral load. Apparently, the Twist Bioscience SARS-Cov-2 panel has the best performance, requiring fewer reads to achieve high coverage. However, further studies comparing all kits and using different samples (clinical and environmental) are needed. It appears that specific probes panels allow high sensitivity and specificity in complex samples to detect SARS-CoV-2 but pan-viral probes panels allow simultaneously various viruses identification.

## 5 Hybridization capture limitations and perspectives

Despite the potential benefits that target enrichment procedures can bring to the study of highly diverse pathogens, there are limitations to their implementation in clinical diagnostics and even in research settings. The main disadvantages of this method are the time required and the high cost of the selective enrichment procedure, which would make it difficult to implement them in low-income settings (Munyuza et al., 2022). In a review of the literature, Hale et al. provide a cost calculator for researchers considering targeted sequencing to use when designing projects and to identify challenges for future development of low-cost sequencing. They estimated the cost of a sample from extraction to NGS to be approximately 65 USD; however, the cost may be lower (e.g., 22 USD) if some modifications are made, for instance, if the probes are diluted and a larger number of samples are pooled during hybridization (Hale et al., 2020). Other modifications to the capture workflow may further reduce costs but depend on the research question to balance considerations such as input DNA quality, sequencing depth, phylogenetic scope, and number of samples. Taken together, these alternatives should enable most research groups to take advantage of the data-generating capacity of targeted sequencing on a reasonable budget (Hale et al., 2020).

In addition, target enrichment procedures are complex (e.g., probe design and synthesis), which requires experienced individuals with knowledge of the different components of the procedures as well as bioinformatics skills. These factors would affect implementation of a target enrichment procedure in a clinical setting, where rapid results are needed, especially in cases where novel pathogens are concerned (Gaudin and Desnues, 2018; Munyuza et al., 2022). Nonetheless, the use of kits and the generation of protocols such as those discussed in this review facilitate implementation of the gene capture approach.

A further point to note is the need for updated probes/panels, mainly in cases where viruses are highly mutagenic. Although the probes can tolerate mutations in the target genome, their performance is significantly influenced by the design, which is contingent upon the timing and number of genomic sequences of the strains used for construction. For instance, probes designed based on early pandemic reference strains may differ from those designed later on. Therefore, it is advisable to promptly upgrade the panels to enhance their performance, allowing them to surpass the originally constructed panels once they have been optimized for the latest variants.

As this review shows, hybridization capture has been widely used in the research setting; however, its potential use in other scenarios or fields is quite significant. For instance, in environmental studies, in which determining species distributions can be extremely difficult. Most environmental DNA or RNA (eDNA, eRNA) studies have employed metabarcoding for species detection and discrimination (Aylagas et al., 2016). As with clinical samples, capture hybridization effectively avoids problems such as primer bias and can substantially increase the yield of target DNA recovered from environmental samples (Bragalini et al., 2014).

In addition to detection in sputum and saliva, SARS-CoV-2 RNA is shed in feces in up to 67 % of infected patients (and is also present in feces of asymptomatic individuals). Consequently, the presence of SARS-CoV-2 RNA in feces indicates that wastewater might be used to control infection rates in a population (Chen et al., 2020; Reynolds et al., 2022). Indeed, wastewater-based epidemiology (WBE) is a useful tool to track virus circulation and variants such as poliovirus (Asghar et al., 2014). Wastewater monitoring was already described in several laboratories around the world from the outset of the COVID-19 pandemic (Reynolds et al., 2022). Interestingly, in The Netherlands, Medema *et al*. identified SARS-CoV-2 RNA in wastewater prior to the first clinical cases (Medema et al., 2020). Considering the changing nature of this virus and the complexity of samples (e.g., air, water, soil, biological materials, and wastes), use of sequencing coupled with hybridization capture represents a superb strategy for SARS-CoV-2 studies, as has been described for other microorganisms (Bragalini et al., 2014; Seeber et al., 2019). The Viral Surveillance Panel kit (Illumina) involves viruses identified as important risks to public health, including SARS-CoV-2; however, to the best of our knowledge, studies employing this methodology have not been reported. In 2021, a Spanish group described use of VirCapSeq Enrichment Kit (multi virus panel) for wastewater samples. However, only eight SARS-CoV-2 contigs were obtained, with a total of 2.03 kb, representing 6.8 % of the genome (Martínez-Puchol et al., 2021). Hence, future studies on this topic should be carried out.

The bioinformatics methods used by the different studies described here were not reviewed in detail, and this could influence the comparisons between studies. Consider reviewing the bioinformatics methods used in the studies of interest.

## 6 Conclusions

Genomic innovations are transforming epidemiology to better characterize, react to and prepare for epidemics and/or pandemics. NGS-based hybrid capture can provide important genetic insights into SARS-CoV-2 and other emerging pathogens and improve surveillance and early detection. Having multiple technologies in the SARS-CoV-2 genome sequencing toolbox should aid us in increasing vigilance for new SARS-CoV-2 variants that might escape vaccines.

In general, studies in which hybrid capture has been used agree that the hybrid capture-based workflow is labor intensive. Furthermore, this approach can be costly and requires a great deal of expertise for library preparation as well as time to generate biotinylated probes from reference genomes. However, target enrichment has proven to be a useful approach for sequencing complex samples, allowing for full sequencing of the viral genome even in specimens with a low viral titre or poor nucleic acid integrity. In addition, this approach is very efficient at discovering new variants, as it is able to capture large regions and even whole genomes. Moreover, it can operate with multiple targets per panel and dozens to hundreds of overlapping capture probes, leading to higher specificity for whole viral genome recovery and greater uniformity of coverage compared to amplicon-based technologies. Given that there are now multiple kits available with probes that target SARS-CoV-2 (and other pathogens), many of the aforementioned problems can be overcome by applying this technique. Therefore, this is an efficient and robust strategy that can be used for NGS-based studies.

## Funding

This work was supported by the European Union (EU), European Funding of Regional Development (FEDER) and Conseil Régional Auvergne under Grant/Award Number: AV0028535. The funders had no role in decision to publish, or preparation of the manuscript.

## Declaration of Competing Interest

The authors declare that they have no known competing financial interests or personal relationships that could have appeared to influence the work reported in this paper.

## Data Availability

No data was used for the research described in the article.

## Appendix Supplementary materials

Image, application 1

## References

[bib0001] Asghar H., Diop O.M., Weldegebriel G., Malik F., Shetty S., Bassioni L.El, Akande A.O., Al Maamoun E., Zaidi S., Adeniji A.J., Burns C.C., Deshpande J., Oberste M.S., Lowther S.A. (2014). Environmental surveillance for polioviruses in the global polio eradication initiative. J. Infect. Dis..

[bib0002] Aylagas E., Borja Á., Irigoien X., Rodríguez-Ezpeleta N. (2016). Benchmarking DNA metabarcoding for biodiversity-based monitoring and assessment. Front. Mar. Sci..

[bib0003] Bokelmann L., Nickel O., Maricic T., Pääbo S., Meyer M., Borte S., Riesenberg S. (2021). Point-of-care bulk testing for SARS-CoV-2 by combining hybridization capture with improved colorimetric LAMP. Nat. Commun..

[bib0004] Bragalini C., Ribière C., Parisot N., Vallon L., Prudent E., Peyretaillade E., Girlanda M., Peyret P., Marmeisse R., Luis P. (2014). Solution hybrid selection capture for the recovery of functional full-length eukaryotic cDNAs from complex environmental samples. DNA Res..

[bib0005] Briese T., Kapoor A., Mishra N., Jain K., Kumar A., Jabado O.J., Ian Lipkina W. (2015). Virome capture sequencing enables sensitive viral diagnosis and comprehensive virome analysis. mBio.

[bib0006] Charre C., Ginevra C., Sabatier M., Regue H., Destras G., Brun S., Burfin G., Scholtes C., Morfin F., Valette M., Lina B., Bal A., Josset L. (2020). Evaluation of NGS-based approaches for SARS-CoV-2 whole genome characterisation. Virus Evol..

[bib0007] Chein Y.H., Davidson N. (1978). RNA:DNA hybrids are more stable than DNA: DNA duplexes in concentrated perchlorate and trichloroacetate solutions. Nucleic. Acids. Res..

[bib0008] Chen Y., Chen L., Deng Q., Zhang G., Wu K., Ni L., Yang Y., Liu B., Wang W., Wei C., Yang J., Ye G., Cheng Z. (2020). The presence of SARS-CoV-2 RNA in the feces of COVID-19 patients. J. Med. Virol..

[bib0009] Credle J.J., Robinson M.L., Gunn J., Monaco D., Sie B., Tchir A., Hardick J., Zheng X., Shaw-Saliba K., Rothman R.E., Eshleman S.H., Pekosz A., Hansen K., Mostafa H., Steinegger M., Larman H.B. (2021). Highly multiplexed oligonucleotide probe-ligation testing enables efficient extraction-free SARS-CoV-2 detection and viral genotyping. Mod. Pathol..

[bib0010] Depledge D.P., Palser A.L., Watson S.J., Lai I.Y.C., Gray E.R., Grant P., Kanda R.K., Leproust E., Kellam P., Breuer J. (2011). Specific capture and whole-genome sequencing of viruses from clinical samples. PLoS One.

[bib0011] Dickson Z.W., Hackenberger D., Kuch M., Marzok A., Banerjee A., Rossi L., Klowak J.A., Fox-Robichaud A., Mossmann K., Miller M.S., Surette M.G., Golding G.B., Poinar H. (2021). Probe design for simultaneous, targeted capture of diverse metagenomic targets. Cell Rep. Methods.

[bib0012] Doddapaneni H., Cregeen S.J., Sucgang R., Meng Q., Qin X., Avadhanula V., Chao H., Menon V., Nicholson E., Henke D., Piedra F.A., Rajan A., Momin Z., Kottapalli K., Hoffman K.L., Sedlazeck F.J., Metcalf G., Piedra P.A., Muzny D.M., Petrosino J.F., Gibbs R.A. (2021). Oligonucleotide capture sequencing of the SARS-CoV-2 genome and subgenomic fragments from COVID-19 individuals. PLoS One.

[bib0013] Ebrahimi S., Khanbabaei H., Abbasi S., Fani M., Soltani S., Zandi M., Najafimemar Z. (2022). CRISPR-Cas system: a promising diagnostic tool for Covid-19. Avicenna J. Med. Biotechnol..

[bib0014] Ellis P., Somogyvári F., Virok D.P., Noseda M., McLean G.R. (2021). Decoding Covid-19 with the SARS-CoV-2 genome. Curr. Genet. Med. Rep..

[bib0016] Gasc C., Peyretaillade E., Peyret P. (2016). Sequence capture by hybridization to explore modern and ancient genomic diversity in model and nonmodel organisms. Nucleic. Acids. Res..

[bib0017] Gasc C., Peyret P. (2018). Hybridization capture reveals microbial diversity missed using current profiling methods. Microbiome.

[bib0018] Gasc C., Peyret P. (2017). Revealing large metagenomic regions through long DNA fragment hybridization capture. Microbiome.

[bib0019] Gaudin M., Desnues C. (2018). Hybrid capture-based next generation sequencing and its application to human infectious diseases. Front. Microbiol..

[bib0020] Gerber Z., Daviaud C., Delafoy D., Sandron F., Alidjinou E.K., Mercier J., Gerber S., Meyer V., Boland A., Bocket L., Olaso R., Deleuze J.F. (2022). A comparison of high-throughput SARS-CoV-2 sequencing methods from nasopharyngeal samples. Sci. Rep..

[bib0021] Ghosh P., Chowdhury R., Hossain M.E., Hossain F., Miah M., Rashid M.U., Baker J., Rahman M.Z., Rahman M., Ma X., Duthie M.S., Wahed A.A.El, Mondal D. (2022). Evaluation of recombinase-based isothermal amplification assays for point-of-need detection of SARS-CoV-2 in resource-limited settings. Int. J. Infec. Dis..

[bib0022] Gire S.K., Goba A., Andersen K.G., Sealfon R.S.G., Park D.J., Kanneh L., Jalloh S., Momoh M., Fullah M., Dudas G., Wohl S., Moses L.M., Yozwiak N.L., Winnicki S., Matranga C.B., Malboeuf C.M., Qu J., Gladden A.D., Schaffner S.F., Yang X., Jiang P.P., Nekoui M., Colubri A., Coomber M.R., Fonnie M., Moigboi A., Gbakie M., Kamara F.K., Tucker V., Konuwa E., Saffa S., Sellu J., Jalloh A.A., Kovoma A., Koninga J., Mustapha I., Kargbo K., Foday M., Yillah M., Kanneh F., Robert W., Massally J.L.B., Chapman S.B., Bochicchio J., Murphy C., Nusbaum C., Young S., Birren B.W., Grant D.S., Scheiffelin J.S., Lander E.S., Happi C., Gevao S.M., Gnirke A., Rambaut A., Garry R.F., Khan S.H., Sabeti P.C. (2014). Genomic surveillance elucidates Ebola virus origin and transmission during the 2014 outbreak. Science.

[bib0023] Gupta R., Sagar P., Priyadarshi N., Kaul S., Sandhir R., Rishi V., Singhal N.K. (2020). Nanotechnology-based approaches for the detection of SARS-CoV-2. Front. Nanotechnol..

[bib0024] Hale H., Gardner E.M., Viruel J., Pokorny L., Johnson M.G. (2020). Strategies for reducing per-sample costs in target capture sequencing for phylogenomics and population genomics in plants. Appl. Plant Sci..

[bib0025] Hartley P.D., Tillett R.L., AuCoin D.P., Sevinsky J.R., Xu Y., Gorzalski A., Pandori M., Buttery E., Hansen H., Picker M.A., Rossetto C.C., Verma S.C. (2021). Genomic surveillance of Nevada patients revealed prevalence of unique SARS-CoV-2 variants bearing mutations in the RdRp gene. J. Genet. Genom..

[bib0026] Hodges E., Xuan Z., Balija V., Kramer M., Molla M.N., Smith S.W., Middle C.M., Rodesch M.J., Albert T.J., Hannon G.J., McCombie W.R. (2007). Genome-wide in situ exon capture for selective resequencing. Nat. Genet..

[bib0015] Fusion Genomics, 2021. Pathogen type/subtype/lineage target target coverage (%) [WWW Document]. URL https://www.fusiongenomics.com/onetest (Accessed December 8, 2022).

[bib0027] Illumina, 2022. How next-generation sequencing can help identify and track SARS-CoV-2 [WWW Document]. URL https://www.nature.com/articles/d42473-020-00120-0 (Accessed November 28, 2022a).

[bib0028] Illumina, n.db. Viral Surveillance Panel | For SARS-COV-2, flu, polio, monkeypox, & more [WWW Document]. URL https://www.illumina.com/products/by-type/sequencing-kits/library-prep-kits/viral-surveillance-panel.html (Accessed December 14, 2022b).

[bib0029] Kim K.W., Deveson I.W., Pang C.N.I., Yeang M., Naing Z., Adikari T., Hammond J.M., Stevanovski I., Beukers A.G., Verich A., Yin S., McFarlane D., Wilkins M.R., Stelzer-Braid S., Bull R.A., Craig M.E., van Hal S.J., Rawlinson W.D. (2021). Respiratory viral co-infections among SARS-CoV-2 cases confirmed by virome capture sequencing. Sci. Rep..

[bib0030] Klempt P., Brož P., Kašný M., Novotný A., Kvapilová K., Kvapil P. (2020). Performance of targeted library preparation solutions for sars-cov-2 whole genome analysis. Diagnostics (Basel).

[bib0031] Kuchinski K.S., Loos K.D., Suchan D.M., Russell J.N., Sies A.N., Kumakamba C., Muyembe F., Mbala Kingebeni P., Ngay Lukusa I., N’Kawa F., Atibu Losoma J., Makuwa M., Gillis A., LeBreton M., Ayukekbong J.A., Lerminiaux N.A., Monagin C., Joly D.O., Saylors K., Wolfe N.D., Rubin E.M., Muyembe Tamfum J.J., Prystajecky N.A., McIver D.J., Lange C.E., Cameron A.D. (2022). Targeted genomic sequencing with probe capture for discovery and surveillance of coronaviruses in bats. eLife.

[bib0032] Maia R., Carvalho V., Faria B., Miranda I., Catarino S., Teixeira S., Lima R., Minas G., Ribeiro J. (2022). Diagnosis methods for COVID-19: a systematic review. Micromachines (Basel).

[bib0033] Malik Y.S., Kumar P., Ansari M.I., Hemida M.G., El Zowalaty M.E., Abdel-Moneim A.S., Ganesh B., Salajegheh S., Natesan S., Sircar S., Safdar M., Vinodhkumar O.R., Duarte P.M., Patel S.K., Klein J., Rahimi P., Dhama K. (2021). SARS-CoV-2 spike protein extrapolation for COVID diagnosis and vaccine development. Front. Mol. Biosci..

[bib0034] Manjur O.H.Bin, Afrad M.H., Khan M.H., Hossain M., Kawser Z., Alam A.N., Banik N., Alam S., Billah M.M., Afreen N., Khanam F., Bhuiyan T.R., Rahman M.Z., Westeel E., Berland J.-L., Komurian-Pradel F., Banu S., Rahman M., Thompson N.R., Qadri F., Shirin T. (2022). Genome sequences of 25 SARS-CoV-2 sublineage B.1.1.529 omicron strains in Bangladesh. Microb. Res. Announc..

[bib0035] Martínez-Puchol S., Itarte M., Rusiñol M., Forés E., Mejías-Molina C., Andrés C., Antón A., Quer J., Abril J.F., Girones R., Bofill-Mas S. (2021). Exploring the diversity of coronavirus in sewage during COVID-19 pandemic: don't miss the forest for the trees. Sci. Tot. Environ..

[bib0036] Medema G., Heijnen L., Elsinga G., Italiaander R., Brouwer A. (2020). Presence of SARS-coronavirus-2 RNA in sewage and correlation with reported COVID-19 prevalence in the early stage of the epidemic in the Netherlands. Environ. Sci. Technol. Lett..

[bib0037] Metsky H.C., Matranga C.B., Wohl S., Schaffner S.F., Freije C.A., Winnicki S.M., West K., Qu J., Baniecki M.L., Gladden-Young A., Lin A.E., Tomkins-Tinch C.H., Ye S.H., Park D.J., Luo C.Y., Barnes K.G., Shah R.R., Chak B., Barbosa-Lima G., Delatorre E., Vieira Y.R., Paul L.M., Tan A.L., Barcellona C.M., Porcelli M.C., Vasquez C., Cannons A.C., Cone M.R., Hogan K.N., Kopp E.W., Anzinger J.J., Garcia K.F., Parham L.A., Ramírez R.M.G., Montoya M.C.M., Rojas D.P., Brown C.M., Hennigan S., Sabina B., Scotland S., Gangavarapu K., Grubaugh N.D., Oliveira G., Robles-Sikisaka R., Rambaut A., Gehrke L., Smole S., Halloran M.E., Villar L., Mattar S., Lorenzana I., Cerbino-Neto J., Valim C., Degrave W., Bozza P.T., Gnirke A., Andersen K.G., Isern S., Michael S.F., Bozza F.A., Souza T.M.L., Bosch I., Yozwiak N.L., Macinnis B.L., Sabeti P.C. (2017). Zika virus evolution and spread in the Americas. Nature.

[bib0038] Miyazato P., Katsuya H., Fukuda A., Uchiyama Y., Matsuo M., Tokunaga M., Hino S., Nakao M., Satou Y. (2016). Application of targeted enrichment to next-generation sequencing of retroviruses integrated into the host human genome. Sci. Rep..

[bib0039] Müller N.F., Wagner C., Frazar C.D., Roychoudhury P., Lee J., Moncla L.H., Pelle B., Richardson M., Ryke E., Xie H., Shrestha L., Addetia A., Rachleff V.M., Lieberman N.A.P., Huang M.L., Gautom R., Melly G., Hiatt B., Dykema P., Adler A., Brandstetter E., Han P.D., Fay K., Ilcisin M., Lacombe K., Sibley T.R., Truong M., Wolf C.R., Boeckh M., Englund J.A., Famulare M., Lutz B.R., Rieder M.J., Thompson M., Duchin J.S., Starita L.M., Chu H.Y., Shendure J., Jerome K.R., Lindquist S., Greninger A.L., Nickerson D.A., Bedford T. (2021). Viral genomes reveal patterns of the SARS-CoV-2 outbreak in Washington State. Sci. Transl. Med..

[bib0040] Munyuza C., Ji H., Lee E.R. (2022). Probe capture enrichment methods for HIV and HCV genome sequencing and drug resistance genotyping. Pathogens.

[bib0041] Nagy-Szakal D., Couto-Rodriguez M., Wells H.L., Barrows J.E., Debieu M., Butcher K., Chen S., Berki A., Hager C., Boorstein R.J., Taylor M.K., Jonsson C.B., Mason C.E., O’Hara N.B. (2021). Targeted hybridization capture of SARS-CoV-2 and metagenomics enables genetic variant discovery and nasal microbiome insights. Microb. Spectr..

[bib0042] Nasir J.A., Kozak R.A., Aftanas P., Raphenya A.R., Smith K.M., Maguire F., Maan H., Alruwaili M., Banerjee A., Mbareche H., Alcock B.P., Knox N.C., Mossman K., Wang B., Hiscox J.A., McArthur A.G., Mubareka S. (2020). A Comparison of Whole Genome sequencing of SARS-CoV-2 using amplicon-based sequencing, random hexamers, and bait capture. Viruses.

[bib0043] No J.S., Kim W.K., Cho S., Lee S.H., Kim J.A., Lee D., Song D.H., Gu S.H., Jeong S.T., Wiley M.R., Palacios G., Song J.W. (2019). Comparison of targeted next-generation sequencing for whole-genome sequencing of Hantaan orthohantavirus in Apodemus agrarius lung tissues. Sci. Rep..

[bib0044] Peeling R.W., Heymann D.L., Teo Y.Y., Garcia P.J. (2022). Diagnostics for COVID-19: moving from pandemic response to control. Lancet.

[bib0045] Perlman S., Netland J. (2009). Coronaviruses post-SARS: update on replication and pathogenesis. Nat. Rev. Microbiol..

[bib0046] Pipoli da Fonseca J., Kornobis E., Turc E., Enouf V., Lemée L., Cokelaer T., Monot M. (2022). Capturing SARS-CoV-2 from patient samples with low viral abundance: a comparative analysis. Sci. Rep..

[bib0047] Pogka V., Papadopoulou G., Valiakou V., Sgouras D.N., Mentis A.F., Karamitros T. (2022). Targeted virome sequencing enhances unbiased detection and genome assembly of known and emerging viruses—the example of SARS-CoV-2. Viruses.

[bib0048] Quick J., Grubaugh N.D., Pullan S.T., Claro I.M., Smith A.D., Gangavarapu K., Oliveira G., Robles-Sikisaka R., Rogers T.F., Beutler N.A., Burton D.R., Lewis-Ximenez L.L., De Jesus J.G., Giovanetti M., Hill S.C., Black A., Bedford T., Carroll M.W., Nunes M., Alcantara L.C., Sabino E.C., Baylis S.A., Faria N.R., Loose M., Simpson J.T., Pybus O.G., Andersen K.G., Loman N.J. (2017). Multiplex PCR method for MinION and Illumina sequencing of Zika and other virus genomes directly from clinical samples. Nat. Protoc..

[bib0049] Radford A.D., Chapman D., Dixon L., Chantrey J., Darby A.C., Hall N. (2012). Application of next-generation sequencing technologies in virology. J. Gen. Virol..

[bib0050] Rehn A., Braun P., Knüpfer M., Wölfel R., Antwerpen M.H., Walter M.C. (2021). Catching SARS-CoV-2 by sequence hybridization: a comparative analysis. mSystems.

[bib0051] Reynolds L.J., Gonzalez G., Sala-Comorera L., Martin N.A., Byrne A., Fennema S., Holohan N., Kuntamukkula S.R., Sarwar N., Nolan T.M., Stephens J.H., Whitty M., Bennett C., Luu Q., Morley U., Yandle Z., Dean J., Joyce E., O’Sullivan J.J., Cuddihy J.M., McIntyre A.M., Robinson E.P., Dahly D., Fletcher N.F., Carr M., De Gascun C., Meijer W.G. (2022). SARS-CoV-2 variant trends in Ireland: wastewater-based epidemiology and clinical surveillance. Sci. Total Environ..

[bib0052] Ribière C., Beugnot R., Parisot N., Gasc C., Defois C., Denonfoux J., Boucher D., Peyretaillade E., Peyret P. (2016). Targeted gene capture by hybridization to illuminate ecosystem functioning. Meth. Mol. Biol..

[bib0053] Sahajpal N.S., Mondal A.K., Njau A., Petty Z., Chen J., Ananth S., Ahluwalia P., Williams C., Ross T.M., Chaubey A., Desantis G., Schroth G.P., Bahl J., Kolhe R. (2021). High-throughput next-generation sequencing respiratory viral panel: a diagnostic and epidemiologic tool for sars-cov-2 and other viruses. Viruses.

[bib0054] Seeber P.A., McEwen G.K., Löber U., Förster D.W., East M.L., Melzheimer J., Greenwood A.D. (2019). Terrestrial mammal surveillance using hybridization capture of environmental DNA from African waterholes. Mol. Ecol. Resour..

[bib0055] Shih S.Y., Bose N., Gonçalves A.B.R., Erlich H.A., Calloway C.D. (2018). Applications of probe capture enrichment next generation sequencing for whole mitochondrial genome and 426 nuclear SNPs for forensically challenging samples. Genes (Basel).

[bib0056] Sulonen A.M., Ellonen P., Almusa H., Lepistö M., Eldfors S., Hannula S., Miettinen T., Tyynismaa H., Salo P., Heckman C., Joensuu H., Raivio T., Suomalainen A., Saarela J. (2011). Comparison of solution-based exome capture methods for next generation sequencing. Genome Biol..

[bib0057] Targeted sequencing of SARS-COV-2: Swift RNA library Kit and arbor biosciences mybaits expert virus panel [WWW Document], 2020. URL www.swiftbiosci.com (Accessed December 8, 2022).

[bib0058] Thomson E., Ip C.L.C., Badhan A., Christiansen M.T., Adamson W., Ansari M.A., Bibby D., Breuer J., Brown A., Bowden R., Bryant J., Bonsall D., Da Silva Filipe A., Hinds C., Hudson E., Klenerman P., Lythgow K., Mbisa J.L., McLauchlan J., Myers R., Piazza P., Roy S., Trebes A., Sreenu V.B., Witteveldt J., Barnes E., Simmonds P. (2016). Comparison of next-generation sequencing technologies for comprehensive assessment of full-length hepatitis C viral genomes. J. Clin. Microbiol..

[bib0059] Twist Bioscience Twist SARS-CoV-2 research panel [WWW Document], n.d. URL https://www.twistbioscience.com/resources/product-sheet/twist-sars-cov-2-research-panel (Accessed December 8, 22).

[bib0060] Wang Y., Wang D., Zhang L., Sun W., Zhang Z., Chen W., Zhu A., Huang Y., Xiao F., Yao J., Gan M., Li F., Luo L., Huang X., Zhang Y., Wong S.san, Cheng X., Ji J., Ou Z., Xiao M., Li M., Li Jiandong, Ren P., Deng Z., Zhong H., Xu X., Song T., Mok C.K.P., Peiris M., Zhong N., Zhao Jingxian, Li Y., Li Junhua, Zhao Jincun (2021). Intra-host variation and evolutionary dynamics of SARS-CoV-2 populations in COVID-19 patients. Genome Med..

[bib0061] Wen S., Sun C., Zheng H., Wang L., Zhang H., Zou L., Liu Z., Du P., Xu X., Liang L., Peng X., Zhang W., Wu J., Yang J., Lei B., Zeng G., Ke C., Chen F., Zhang X. (2020). High-coverage SARS-CoV-2 genome sequences acquired by target capture sequencing. J. Med. Virol..

[bib0062] Wylezich C., Calvelage S., Schlottau K., Ziegler U., Pohlmann A., Höper D., Beer M. (2021). Next-generation diagnostics: virus capture facilitates a sensitive viral diagnosis for epizootic and zoonotic pathogens including SARS-CoV-2. Microbiome.

[bib0063] Xiao M., Liu X., Ji J., Ji J., Ji J., Li M., Li M., Li M., Li Jiandong, Li Jiandong, Li Jiandong, Yang L., Sun W., Sun W., Sun W., Ren P., Ren P., Yang G., Zhao J., Zhao J., Liang T., Liang T., Ren H., Chen T., Zhong H., Song W., Song W., Wang Y., Deng Z., Deng Z., Zhao Y., Zhao Y., Ou Z., Ou Z., Wang D., Wang D., Cai J., Cheng X., Cheng X., Cheng X., Feng T., Wu H., Gong Y., Yang H., Yang H., Wang J., Wang J., Xu X., Xu X., Zhu S., Zhu S., Chen F., Chen F., Zhang Y., Chen W., Chen W., Li Y., Li Junhua, Li Junhua, Li Junhua (2020). Multiple approaches for massively parallel sequencing of SARS-CoV-2 genomes directly from clinical samples. Genome Med..

[bib0064] Zhan S.H., Alamouti S.M., Daneshpajouh H., Kwok B.S., Lee M.H., Khattra J., Houck H.J., Rand K.H. (2021). Target capture sequencing of SARS-CoV-2 genomes using the ONETest Coronaviruses Plus. Diagn. Microbiol. Infect. Dis..

[bib0065] Zhou P., Yang X.Lou, Wang X.G., Hu B., Zhang L., Zhang W., Si H.R., Zhu Y., Li B., Huang C.L., Chen H.D., Chen J., Luo Y., Guo H., Jiang R.Di, Liu M.Q., Chen Y., Shen X.R., Wang X., Zheng X.S., Zhao K., Chen Q.J., Deng F., Liu L.L., Yan B., Zhan F.X., Wang Y.Y., Xiao G.F., Shi Z.L. (2020). A pneumonia outbreak associated with a new coronavirus of probable bat origin. Nature.

