# Supplementary material for: Applying targeted gene hybridization capture to viruses with a focus to SARS-CoV-2
Source: Virus Res. 2023 Dec 16;340:199293. doi: 10.1016/j.virusres.2023.199293 (PMC10767490; doi:10.1016/j.virusres.2023.199293)
Supplement: Supplementary file 1 [file mmc1.pdf]

Table S1. list of viruses targeted by hybridization capture kits

VirCapSeq-VERT	Respiratory Virus Oligo Panel	Viral survillance Panel	Respiratory Pathogen ID/AMR Enrichment Panel Kit	Twist Respiratory Virus Research Panel	ONETestTM Coronaviruses Plus	VirBaits Wylezich et al 2021
Adenoviridae	Human coronavirus 229E	Adenovirus	Achromobacter denitrificans	Human adenovirus	Coronaviruses	African horse sickness virus
Alloherpesviridae	Human coronavirus NL63	Aichivirus	Bartonella henselae	Human bocavirus	Influenza virus	African swine fever virus
Alphacoronavirus	Human coronavirus OC43	Astrovirus	Cardiobacterium hominis	Human coronavirus	Respiratory syncytial virus	Bovine herpesvirus 1
Alphaherpesvirinae	Human coronavirus HKU1	Chapare virus	Elizabethkingia meningoseptica	Human enterovirus	Human metapneumovirus	Lumpy skin disease virus
Alphanodavirus	SARS-CoV-2	Chikungunya virus	Haemophilus influenzae	Human metapneumovirus	Parainfluenza virus 1 and 3	Monkeypox virus
Alphapapillomavirus	Human adenovirus B1	Coronavirus-229E	Mycobacterium gordonae	Human parainfluenza virus	Parainfluenza virus 2 and 4	Sheeppox virus/goatpox virus
Alphapermutotetravirus	Human adenovirus C2	Coronavirus-HKU1	Achromobacter xylosoxidans	Human rhinovirus	Enterovirus/ rhinovirus	Suid alphaherpesvirus 1
Alpharetrovirus	Human adenovirus E4	Coronavirus-OC43	Bartonella quintana	Human rubulavirus	Parechovirus	Bluetongue virus
Alphatorquevirus	Human bocavirus 1 (Primate bocaparvovirus 1 isolate st2)	Coronavirus-NL63	Cardiobacterium valvarum	Influenza B	Adenovirus	Epizootic hemorrhagic disease virus
Alphavirus	Human bocavirus 2c PK isolate PK-5510	Coxsackievirus	Enterobacter cloacae	Influenza H1N1 (2009)	Bocavirus	Borna disease virus/Bornaviridae
Amdoparvovirus	Human bocavirus 3	Crimean-congo haemorrhagic fever virus	Haemophilus parahaemolyticus	Measles		Bovine leukemia virus
Anelloviridae	Human parainfluenza virus 1	Dengue virus 1	Mycobacterium kansasii	Influenza H3N2		Bovine viral diarrhea virus 1, 2
Aphthovirus	Human parainfluenza virus 2	Dengue virus 2	Acinetobacter baumannii	MERS		Classical swine fever virus
Aquabirnavirus	Human parainfluenza virus 3	Dengue virus 3	Bordetella bronchiseptica	Mumps		Crimean-Congo hemorrhagic fever virus
Aquamavirus	Human parainfluenza virus 4a	Dengue virus 4	Chlamydia pneumoniae	Respiratory syncytial		Ebolavirus
Aquaparamyxovirus	Human metapneumovirus (CAN97-83)	Eastern equine encephalitis virus	Enterococcus faecalis	virus (A & B)		Equine encephalitis virus
Aquareovirus	Respiratory syncytial virus (type A)	Ebola virus	Haemophilus parainfluenzae	Rubella		Equine infectious anemia virus
Arenaviridae	Human Respiratory syncytial virus 9320 (type B)	Enterovirus	Mycobacterium malmoense	SARS		Foot-and-mouth disease virus
Arenavirus	Influenza A virus (A/Puerto Rico/8/1934(H1N1))	Guanarito virus	Acinetobacter lwoffii	SARS-CoV-2		Hendra henipavirus
Arteriviridae	Influenza A virus (A/Korea/426/1968(H2N2))	Hantavirus	Bordetella hinzii			Influenza A virus
Arterivirus	Influenza A virus (A/New York/392/2004(H3N2))	Hendra henipavirus	Chlamydia psittaci			Newcastle disease virus

Table S1. list of viruses targeted by hybridization capture kits

Asfarviridae	Influenza A virus (A/goose/Guangdong/1/1996(H5N1))	Hepatitis A virus	Enterococcus faecium			Nipah henipavirus
Asfivirus	Human bocavirus 4 NI strain HBoV4-NI-385	Hepatitus B virus	Haemophilus pittmaniae			Rabies lyssavirus
Astroviridae	KI polyomavirus Stockholm 60	Hepatitus C virus	Mycobacterium parascrofulaceum			Rift Valley fever virus
Atadenovirus	WU Polyomavirus	Hepatitus E virus	Acinetobacter nosocomialis			Rinderpest virus
Aurivirus	Human parechovirus type 1 PicoBank/HPeV1/a	Human Immunodeficiency Virus 1	Bordetella holmesii			SARS-CoV-2
Avastrovirus	Human parechovirus 6	Human Immunodeficiency Virus 2	Chlamydia trachomatis			Schmallenberg virus
Aveparvovirus	Human rhinovirus A89	Influenza A Virus	Escherichia coli			Small ruminant morbillivirus
Aviadenovirus	Human rhinovirus C (strain 024)	Influenza B Virus	Hafnia alvei			Swine vesicular disease virus
Avibirnavirus	Human rhinovirus B14	Japanese encephalitis virus	Mycobacterium scrofulaceum			Vesicular stomatitis Indiana virus
Avihepadnavirus	Human enterovirus C104 strain: AK11	Junin virus	Acinetobacter pittii			West Nile virus
Avihepatovirus	Human enterovirus C109 isolate NICA08-4327	Kyasanur Forest disease virus	Bordetella parapertussis			
Avipoxvirus	Influenza A virus (A/Zhejiang/DTID-ZJU01/2013(H7N9))	Lassa fever virus	Chromobacterium violaceum			
Avisivirus	Influenza A virus (A/Hong Kong/1073/99(H9N2))	Lujo hemorrphagic fever virus	Eubacterium brachy			
Avulavirus	Influenza A virus (A/Texas/50/2012(H3N2))	Machupo virus	Klebsiella variicola			
Bafinivirus	Influenza A virus (A/Michigan/45/2015(H1N1))	Marburg virus	Mycobacterium szulgai			
Batrachovirus	Influenza B virus (B/Lee/1940)	MERS-CoV	Actinomyces graevenitzii			
Betacoronavirus	Influenza B virus (B/Wisconsin/01/2010)	Metapneumovirus	Bordetella pertussis			
Betaherpesvirinae	Influenza B virus (B/Brisbane/60/2008)	Monkeypox virus	Citrobacter freundii			
Betanodavirus	Influenza B virus (B/Colorado/06/2017)	Nipah virus	Eubacterium limosum			
Betapapillomavirus	Influenza B virus (B/Washington/02/2019)	Norovirus	Kytococcus sedentarius			
Betaretrovirus		Omsk hemorrhagic fever virus	Mycobacterium tuberculosis			

Table S1. list of viruses targeted by hybridization capture kits

Betatorquevirus		Oncolytic human papillomavirus	Actinomyces israelii			
Birnaviridae		Parainfluenza virus	Bordetella petrii			
Blosnavirus		Parechovirus	Citrobacter koseri			
Bocaparvovirus		Parvovirus	Eubacterium nodatum			
Bornaviridae		Poliovirus	Leclercia adecarboxylata			
Bornavirus		Polymavirus	Mycobacterium xenopi			
Bracorhabdovirus		Respiratory syncytial virus	Actinomyces meyeri			
Bunyaviridae		Rhinovirus	Brucella abortus			
Caliciviridae		Rift Valley fever virus	Corynebacterium diphtheriae			
Capripoxvirus		Rotavirus	Finegoldia magna			
Cardiovirus		Rubella virus	Legionella anisa			
Cervidpoxvirus		Sabia virus	Mycobacteroides abscessus (Mycobacterium abscessus)			
Chipapillomavirus		Salivirus	Actinomyces naeslundii			
Chloriridovirus		Sapovirus	Brucella canis			
Chordopoxvirinae		SARS-COV	Corynebacterium jeikeium			
Circoviridae		SARS-COV-2	Francisella tularensis			
Circovirus		Tick-borne encephalitis virus	Legionella feeleii			
Coltivirus		Torque Teno virus	Mycobacteroides chelonae (Mycobacterium chelonae)			
Copiparvovirus		Variola virus	Actinomyces odontolyticus			
Coronaviridae		Venezuelan equine encephalitis virus	Brucella melitensis			
Coronavirinae		West Nile virus	Corynebacterium propinquum			
Cosavirus		Western equine encephalitis virus	Fusobacterium necrophorum			
Crocodylidpoxvirus		Yellow fever virus	Legionella longbeachae			
Cuevavirus		Zika virus	Mycobacteroides immunogenum (Mycobacterium immunogenum)			
Cyprinivirus			Aeromonas caviae			

Table S1. list of viruses targeted by hybridization capture kits

Cytomegalovirus			Brucella suis			
Cytorhabdovirus			Corynebacterium pseudodiphtheriticum			
Deltacoronavirus			Fusobacterium nucleatum			
Deltapapillomavirus			Legionella maceachernii			
Deltaretrovirus			Mycoplasma pneumoniae			
Deltatorquevirus			Aeromonas hydrophila			
Deltavirus			Burkholderia cepacia complex			
Dengue virus group			Corynebacterium pseudotuberculosis			
Densovirinae			Gemella haemolysans			
Dependoparvovirus			Legionella pneumophila			
Dicipivirus			Neisseria flavescens			
Dinornavirus			Aeromonas sobria			
Dyodeltapapillomavirus			Burkholderia gladioli			
Dyoepsilonpapillomavirus			Corynebacterium striatum			
Dyoetapapillomavirus			Gemella morbillorum			
Dyoiotapapillomavirus			Legionella wadsworthii			
Dyokappapapillomavirus			Neisseria lactamica			
Dyolambdapapillomavirus			Aeromonas veronii			
Dyomupapillomavirus			Burkholderia glumae			
Dyonupapillomavirus			Corynebacterium ulcerans			
Dyoomikronpapillomavirus			Gordonia araii			
Dyopipapillomavirus			Leptospira interrogans			
Dyorhopapillomavirus			Neisseria meningitidis			
Dyosigmapapillomavirus			Aggregatibacter actinomycetemcomitans			
Dyothetapapillomavirus			Burkholderia mallei			
Dyoxipapillomavirus			Coxiella burnetii			
Dyozetapapillomavirus			Gordonia bronchialis			
Ebolavirus			Leptotrichia buccalis			

Table S1. list of viruses targeted by hybridization capture kits

Enterovirus			Neisseria mucosa			
Entomopoxvirinae			Aggregatibacter aphrophilus			
Ephemerovirus			Burkholderia pseudomallei			
Epsilonretrovirus			Cronobacter sakazakii			
Epsilontorquevirus			Haemophilus haemolyticus			
Equine lentivirus group			Listeria monocytogenes			
Erbovirus			Nocardia abscessus			
Erythroparvovirus			Arcanobacterium haemolyticum			
Etapapillomavirus			Burkholderia thailandensis			
Etatorquevirus			Delftia acidovorans			
Ferlavirus			Kingella kingae			
Filoviridae			Moraxella catarrhalis			
Flaviviridae			Nocardia arthritidis			
Flavivirus			Bacillus anthracis			
Gallivirus			Campylobacter concisus			
Gammacoronavirus			Dialister pneumosintes			
Gammaherpesvirinae			Klebsiella aerogenes (Enterobacter aerogenes)			
Gammapapillomavirus			Moraxella osloensis			
Gammaretrovirus			Nocardia beijingensis			
Gammatorquevirus			Bacillus cereus			
Gyrovirus			Capnocytophaga gingivalis			
Hantavirus			Dolosigranulum pigrum			
Henipavirus			Klebsiella oxytoca			
Hepacivirus			Morganella morganii			
Hepadnaviridae			Nocardia brasiliensis			
Hepatovirus			Bacillus thuringiensis			
Hepeviridae			Capnocytophaga leadbetteri			
Hepevirus			Eikenella corrodens			

Table S1. list of viruses targeted by hybridization capture kits

Herpesvirales			Klebsiella pneumoniae			
Herpesviridae			Mycobacterium avium complex			
Hunnivirus			Nocardia cyriacigeorgica			
Ichtadenovirus			Bacteroides fragilis			
Ictalurivirus			Capnocytophaga sputigena			
Iltovirus			Elizabethkingia anophelis			
Influenzavirus D			Klebsiella quasipneumoniae			
Intracisternal A-particles			Mycobacterium fortuitum			
Iotatorquevirus			Nocardia farcinica			
Iridoviridae			Nocardia nova			
Iridovirus			Pediococcus acidilactici			
Isavirus			Pseudomonas aeruginosa			
Japanese encephalitis virus group			Serratia marcescens			
Kappapapillomavirus			Streptococcus intermedius			
Kappatorquevirus			Ureaplasma urealyticum			
Kobuvirus			Nocardia otitidiscaviarum			
Kokobera virus group			Peptostreptococcus anaerobius			
Lagovirus			Pseudomonas fluorescens			
Lambdapapillomavirus			Shewanella putrefaciens			
Lambdatorquevirus			Streptococcus mitis			
Lentivirus			Veilonella parvula			
Leporipoxvirus			Nocardia transvalensis			
Lymphocryptovirus			Prevotella buccae			
Lymphocystivirus			Pseudomonas stutzeri			
Lyssavirus			Slackia exigua			
Macavirus			Streptococcus pneumoniae			
Malacoherpesviridae			Williamsia muralis			
Mamastrovirus			Nocardia veterana			

Table S1. list of viruses targeted by hybridization capture kits

Marburgvirus			Prevotella intermedia			
Mardivirus			Ralstonia pickettii			
Mastadenovirus			Sphingomonas paucimobilis			
Megalocytivirus			Streptococcus pyogenes			
Megrivirus			Yersinia enterocolitica			
Metapneumovirus			Ochrobactrum anthropi			
Mischivirus			Prevotella melaninogenica			
Modoc virus group			Raoultella ornithinolytica			
Molluscipoxvirus			Staphylococcus aureus			
Mononegavirales			Tatlockia micdadei (Legionella micdadei)			
Morbillivirus			Yersinia pestis			
Mosavirus			Orientia tsutsugamushi			
mosquito-borne viruses			Prevotella pleuritidis			
Mupapillomavirus			Raoultella planticola			
Muromegalovirus			Stenotrophomonas maltophilia			
Nairovirus			Treponema denticola			
Nebovirus			Pandoraea pulmonicola			
Negevirus			Proteus mirabilis			
Nidovirales			Rhodococcus hoagii			
Nodaviridae			Streptococcus agalactiae			
Norovirus			Tropheryma whipplei			
Novirhabdovirus			Pantoea agglomerans			
Ntaya virus group			Proteus penneri			
Nucleorhabdovirus			Rickettsia rickettsii			
Nupapillomavirus			Streptococcus anginosus			
Nyamiviridae			Tsukamurella pulmonis			
Nyavirus			Parvimonas micra			
Omegapapillomavirus			Proteus vulgaris			

Table S1. list of viruses targeted by hybridization capture kits

Orbivirus			Rothia mucilaginosa			
Orthobunyavirus			Streptococcus constellatus			
Orthohepadnavirus			Tsukamurella tyrosinosolvens			
Orthomyxoviridae			Pasteurella multocida			
Orthopoxvirus			Providencia stuartii			
Orthoreovirus			Salmonella enterica			
Orthoretrovirinae			Streptococcus dysgalactiae			
Oscivirus			Ureaplasma parvum			
Ostreavirus			Coxsackievirus A HHV6			
Papillomaviridae			Human Coronavirus NL63			
Paramyxoviridae			Human parechovirus			
Paramyxovirinae			Influenza C virus			
Parapoxvirus			Rhinovirus A			
Parechovirus			Coxsackievirus B			
Parvoviridae			Human adenovirus B			
Parvovirinae			Human Coronavirus OC43			
Pasivirus			Influenza A virus (H1N1)			
Passerivirus			Measles Virus			
Pegivirus			Rhinovirus B			
Percavirus			Cytomegalovirus (CMV)			
Perhabdovirus			Human adenovirus C			
Pestivirus			Human metapneumovirus			
Phipapillomavirus			Influenza A virus (H3N2)			
Phlebovirus			MERS coronavirus (MERSCoV)			
Picobirnaviridae			Rhinovirus C EBV			
Picobirnavirus			Human adenovirus E			
Picornavirales			Human parainfluenza virus 1			
Picornaviridae			Influenza A virus (H5N1)			
Pipapillomavirus			Mumps virus			

Table S1. list of viruses targeted by hybridization capture kits

Pneumovirinae			Rubella virus			
Pneumovirus			Enterovirus A71			
Polyomaviridae			Human bocavirus 1			
Polyomavirus			Human parainfluenza virus 2			
Poxviridae			Influenza A virus (H7N9)			
Proboscivirus			Parvovirus B19			
Protoparvovirus			SARS coronavirus			
Psipapillomavirus			Enterovirus D68			
Quadrivirus			Human Coronavirus 229E			
Quaranjavirus			Human parainfluenza virus 3			
Ranavirus			Influenza A virus (H9N2)			
Recovirus			Respiratory Syncytial Virus A			
Reoviridae			SARS-CoV-2 (2019-nCoV)			
Respirovirus			Herpes simplex virus 1 (HSV-1)			
Retroviridae			Human Coronavirus HKU1			
Rhabdoviridae			Human parainfluenza virus 4			
Rhadinovirus			Influenza B virus			
Rhopapillomavirus			Respiratory Syncytial Virus B			
Rio Bravo virus group			Varicella-zoster virus (HHV-3)			
Rosavirus			Alternaria alternata			
Roseolovirus			Blastomyces dermatitidis			
Rotavirus			Curvularia lunata			
Rubivirus			Lomentospora prolificans (Scedosporium prolificans)			
Rubulavirus			Pneumocystis jirovecii			
Salivirus			Sarocladium kiliense (Acremonium kiliense)			
Salmonivirus			Alternaria infectoria			
Sapelovirus			Candida auris			
Sapovirus			Exophiala dermatitidis			

Table S1. list of viruses targeted by hybridization capture kits

Scutavirus			Microascus cinereus (Scopulariopsis cinereus)			
Seaborne tick-borne virus group			Purpureocillium lilacinum			
Seadornavirus			Scedosporium apiospermum			
Sedoreovirinae			Apophysomyces elegans			
Senecavirus			Cladophialophora bantiana			
Siadenovirus			Fusarium oxysporum			
Sigmapapillomavirus			Microascus cirrosus (Scopulariopsis paisii)			
Sigmavirus			Rasamsonia aegroticola			
Simplexvirus			Schizophyllum commune			
Spinareovirinae			Aspergillus flavus			
Sprivivirus			Coccidioides immitis			
Spumaretrovirinae			Fusarium proliferatum			
Spumavirus			Microasucus paisii (Scopulariopsis brumptii)			
Suipoxvirus			Rasamsonia argillacea			
Taupapillomavirus			Scopulariopsis brevicaulis			
Teschovirus			Aspergillus fumigatus			
Tetraparvovirus			Coccidioides posadasii			
Thetapapillomavirus			Fusarium solani			
Thetatorquevirus			Mucor circinelloides			
Thogotovirus			Rhizomucor pusillus			
Tibrovirus			Sporothrix schenckii			
tick-borne encephalitis virus group			Aspergillus nidulans			
Togaviridae			Cryptococcus gattii			
Torovirinae			Fusarium verticillioides			
Torovirus			Mucor indicus			
Tremovirus			Rhizopus azygosporus			
Tupavirus			Syncephalastrum racemosum			

Table S1. list of viruses targeted by hybridization capture kits

Upsilonpapillomavirus			Aspergillus niger			
Varicellovirus			Cryptococcus neoformans			
Vesiculovirus			Histoplasma capsulatum			
Vesivirus			Mucor racemosus			
Yatapoxvirus			Rhizopus microsporus			
Yellow fever virus group			Talaromyces marneffei			
Zetapapillomavirus			Aspergillus terreus			
Zetatorquevirus			Cunninghamella bertholletiae			
			Lichtheimia corymbifera			
			Paecilomyces variotii			
			Rhizopus oryzae			
			Trichosporon asahii			
			Aspergillus versicolor			
			Curvularia geniculata			
			Lichtheimia ramosa			
			Paracoccidioides brasiliensis			
			Saksenaea vasiformis			
